# Supplementary material for: A Newly Developed Method-Based Xanthine Oxidoreductase Activities in Various Human Liver Diseases
Source: Biomedicines. 2023 May 14;11(5):1445. doi: 10.3390/biomedicines11051445 (PMC10216503; doi:10.3390/biomedicines11051445)
Supplement: Supplementary file 1 [file biomedicines-11-01445-s001.zip › biomedicines-2306958-supplementary.pdf]

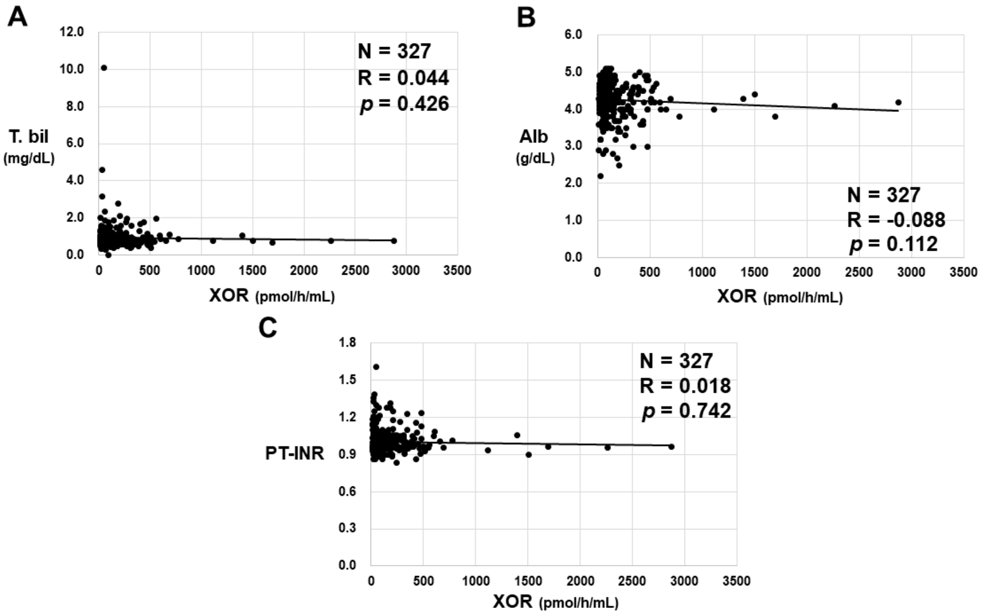

Supplemental Figure S1. The relationship between plasma XOR activities and the levels of parameters of liver tests: (**A**) total bilirubin (T. bil), (**B**) albumin (Alb) and (**C**) prothrombin time-international normalized ratio (PT-INR). The correlation between two values was analyzed using Spearman's rank-correlation coefficient. N; the number of subjects, R; correlation coefficient.

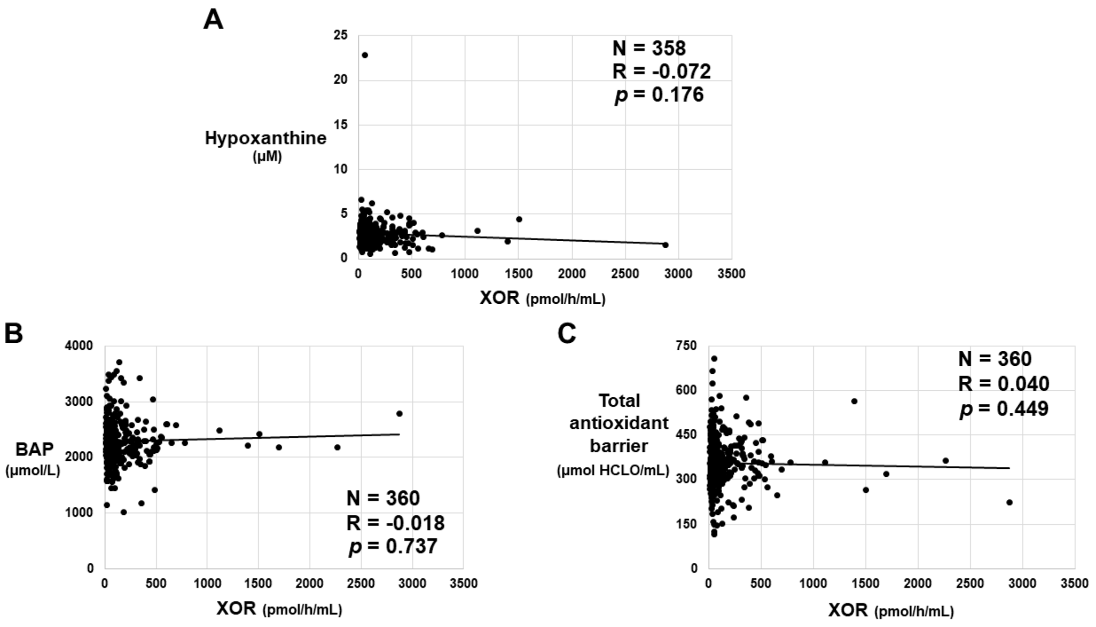

Supplemental Figure S2. The relationship between plasma XOR activities and the values of the purine metabolism-related marker (A) hypoxanthine, the oxidative stress markers (B) biological antioxidant potential (BAP) and (C) total antioxidant barrier. The correlation between two values was analyzed using Spearman's rank-correlation coefficient. N; the number of subjects, R; correlation coefficient.
